# Protein structure prediction based on particle swarm optimization and tabu search strategy

**DOI:** 10.1186/s12859-022-04888-4

**Published:** 2022-08-23

**Authors:** Yu Shuchun, Li Xianxiang, Tian Xue, Pang Ming

**Affiliations:** 1 China Higher Educational Key Laboratory for Measuring and Control Technology and Instrumentation of Heilongjiang Province, Harbin University of Science and Technology, Harbin, 150080 China; 2 Logistics School, Beijing Wuzi University, Beijing, 101149 China; 3 College of Automation, Harbin Engineering University, Harbin, 150001 Heilongjiang China

**Keywords:** Protein, Stable structure, Prediction, Fusion algorithm

## Abstract

**Background:**

The stability of protein sequence structure plays an important role in the prevention and treatment of diseases.

**Results:**

In this paper, particle swarm optimization and tabu search are combined to propose a new method for protein structure prediction. The experimental results show that: for four groups of artificial protein sequences with different lengths, this method obtains the lowest potential energy value and stable structure prediction results, and the effect is obviously better than the other two comparison methods. Taking the first group of protein sequences as an example, our method improves the prediction of minimum potential energy by 127% and 7% respectively.

**Conclusions:**

Therefore, the method proposed in this paper is more suitable for the prediction of protein structural stability.

## Background

Bioinformatics uses computer software and hardware technology to calculate, analyze and mine biological data, which greatly improves the efficiency of biological data information solution, and is of great significance to more accurately grasp the operation law of biological structure [1, 2].

Protein molecules constitute the tissues and organs of the human body. The spatial structure of white matter determines the life function of protein, and then affects and even determines the life activities of the human body. Many diseases occur because the structure of protein molecules is destroyed or mutated, which makes the protein lose its stable state [3, 4].

According to the principle of thermodynamics, when the potential energy value of protein reaches the lowest, the protein structure is in the most stable state. Therefore, using computer algorithm to predict protein stable structure has become an important subject in the field of bioinformatics. The analysis methods of protein structure prediction can be divided into three categories: comparative modeling method, reverse folding method and ab initio prediction method [5–7]. The comparison modeling method and the reverse folding method need the known protein structure as the template, while the ab initio prediction method does not. Therefore, ab initio prediction method can predict the protein structure with unknown information on the premise of building a targeted energy function model [8].

For protein structure modeling, HP lattice model and ab non lattice model are the most commonly used. For HP model, Zhou uses Monte Carlo algorithm and genetic algorithm to solve it. Monte Carlo algorithm uses random number to generate a molecular structure, and then calculates the energy value of the molecular structure. Through continuous iteration, a molecular structure with the lowest energy value is obtained, which is regarded as the real structure of the protein sequence to be tested [9]. During the implementation of Monte Carlo method, due to the random generation of molecular structure, great uncertainty will be caused, which will consume a lot of calculation time and affect the efficiency of protein structure prediction. Therefore, pal added the idea of genetic algorithm to the Monte Carlo method, and the prediction efficiency was improved [10]. Biehn improved Monte Carlo method to chain growth algorithm by increasing and decreasing branches [11]. AB non lattice model can better reflect the real characteristics of protein, and consider the hydrophobicity and hydrophilicity of amino acids in protein structure. Based on AB non lattice model, a variety of intelligent algorithms are used to predict protein structure, including neural network, simulated annealing algorithm, tabu search algorithm, genetic algorithm, particle swarm optimization algorithm and so on. Ting used neural network to predict protein structure, and obtained the lowest energy value and corresponding spatial structure of each sequence [12]. Matsuno improved the simulated annealing algorithm to solve the protein structure prediction problem of two-dimensional AB non lattice model [13]. Makigaki mixed genetic algorithm and tabu search algorithm to calculate the protein structure prediction problem of 3D AB non lattice model [14]. Rakhshani combined deep learning and numerical optimization methods to analyze the optimal morphology of protein structure [15]. Xia proposed a multi peak sampling method, which can also be used to study the stability of protein structure [16].

To sum up, there are many methods in the field of protein structure prediction, but the accurate prediction of the lowest potential energy point of protein stable structure is still a difficult problem. In addition, the amount of data corresponding to protein is huge, which requires high convergence speed and accuracy of the algorithm. Therefore, this paper improves the particle swarm optimization algorithm and integrates tabu search strategy to improve its performance in protein structure prediction.

## Results

In order to verify the effectiveness of the protein structure prediction method proposed in this paper, the next experimental study is carried out. The computer CPU used in the experiment is Intel dual core, single core dominant frequency is 3.60ghz, memory size is 16 GB. The operating system of the computer is windows 10.

In the experiment, the parameters of this algorithm are: the total number of particles is 260, and the upper limit of iterations is 800, two learning factors $\lambda_{1} = \lambda_{2} = 2.0$, scale factor $K = 0.93$, number of neighborhood solutions $L = 40$, number of candidate solutions $L_{C} = 6$.

In the experiment, in order to compare with the algorithm in this paper, particle swarm optimization algorithm and tabu search method are also selected as the control method in the experiment.

**Fig. 1:**
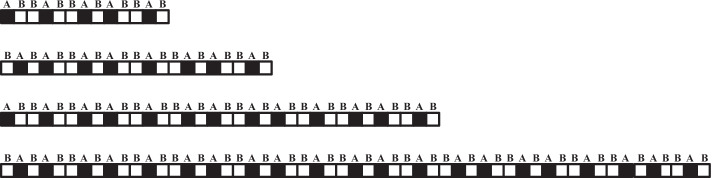
Four artificial protein sequences

In the experiment, the experimental subjects selected the artificial protein sequences commonly used in the experimental standards, and were divided into four groups. The first group of artificial protein sequence is 13, which contains 5 hydrophilic amino acids (a) and 8 hydrophobic amino acids (b); The second group of artificial proteins is 21 in length, including 8 hydrophilic amino acids (a) and 13 hydrophobic amino acids (b); The length of the third group of artificial proteins is 34, including 13 hydrophilic amino acids (a) and 21 hydrophobic amino acids (b); The length of the fourth group of artificial proteins is 55, including 21 hydrophilic amino acids (a) and 34 hydrophobic amino acids (b). The amino acid configuration of the four artificial protein sequences is as Fig. 1.

In simulation experiments, the stability of protein structure is generally judged by potential energy value. The lower the potential energy value, the more stable the protein structure is. Therefore, the comparison of potential energy becomes the key criterion for the stability of protein structure.

According to the above four groups of artificial protein sequences, the mathematical models were constructed according to tabu search method, particle swarm optimization algorithm and the algorithm in this paper, and the lowest potential energy value was predicted. The results are shown in Table 1.

**Table 1:** Minimum potential energy values obtained by three methods

	Tabu search method	Particle swarm optimization method	Our method
The first series	− 1.456	− 3.083	− 3.301
The second series	− 2.839	− 5.412	− 6.299
The third series	− 4.851	− 8.709	− 10.021
The fourth series	− 7.032	− 13.693	− 16.587

It can be seen from the experimental results in Table 1 that the minimum potential energy values of the four groups of artificial protein sequences obtained by this algorithm are significantly lower than those obtained by Tabu search method and particle swarm optimization algorithm. It also shows that more stable protein structure can be obtained by using this algorithm.

## Discussion

**Fig. 2:**
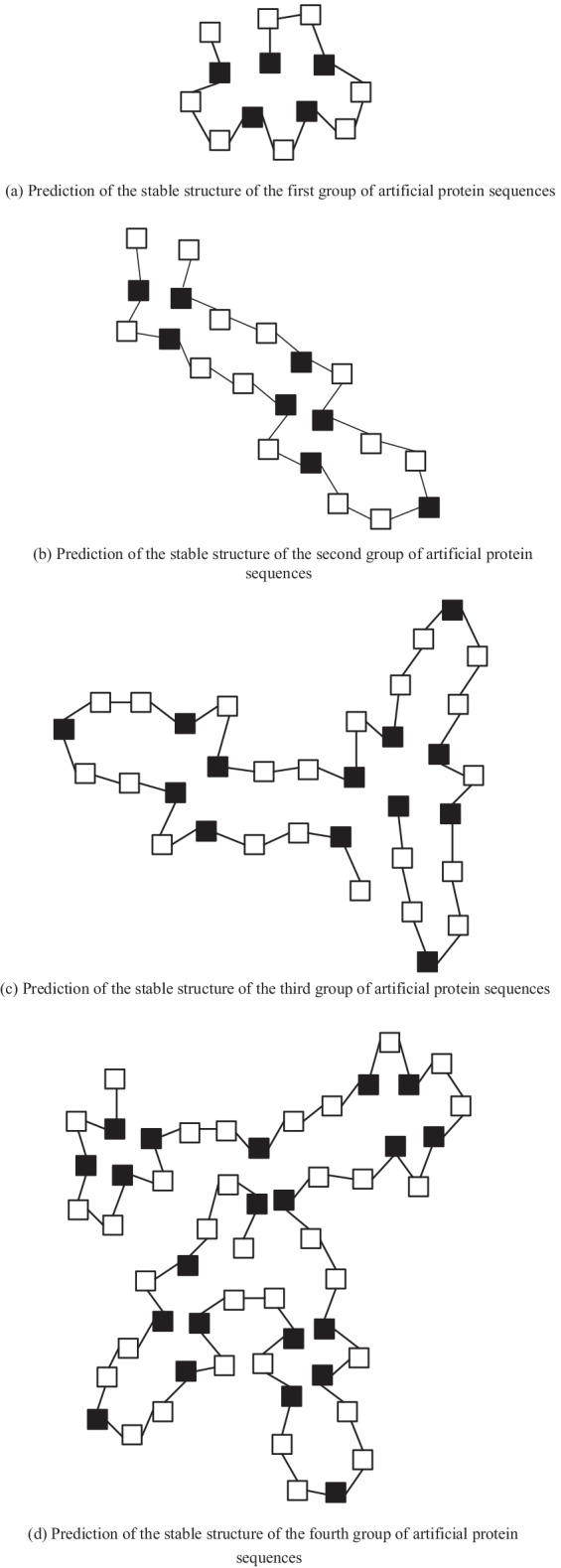
Prediction results of stable structure of four artificial protein sequences.

Furthermore, the stable structures of the above four artificial protein sequences were predicted, and the results are shown in Fig. 2.

## Conclusions

The research on the stability of protein sequence structure is an important content in the field of bioinformatics. In order to solve this problem, a new protein structure prediction method is proposed by combining tabu search and particle swarm optimization. This method fully considers the dependence of tabu search method on the initial solution. Firstly, particle swarm optimization algorithm is used to obtain the initial solution, and then tabu search method is used to construct neighborhood function, candidate solution set, tabu list and tabu criterion. The fusion of the two algorithms is used to complete the prediction of protein structure. In the experiment, four groups of artificial protein sequences were selected. Firstly, the AB non lattice model was used to build the model, and then three methods were used to calculate the lowest potential energy. Experimental results show that, compared with tabu search and particle swarm optimization, this algorithm can get lower potential energy value of protein sequence and predict more reasonable protein stable structure. Taking the first group of protein sequences as an example, the lowest potential energy calculated by this method is further reduced by 127% compared with tabu search method and 7% compared with particle swarm optimization algorithm.

## Methods

Protein structure prediction is actually a complex polynomial non deterministic problem, namely NP complete problem. In this paper, ab initio prediction is selected as a method to solve the problem of protein structure prediction. Therefore, it is necessary to determine the simplified model of protein structure. By constructing a simplified model, the relationship between protein structure and energy is simulated as a potential energy function. Then, according to the thermodynamic hypothesis, when the potential energy function of protein is the lowest, it is the most stable structure of protein.

At present, there are two commonly used simplified protein structure models, one is HP lattice model, the other is AB non lattice model.

**Fig. 3:**
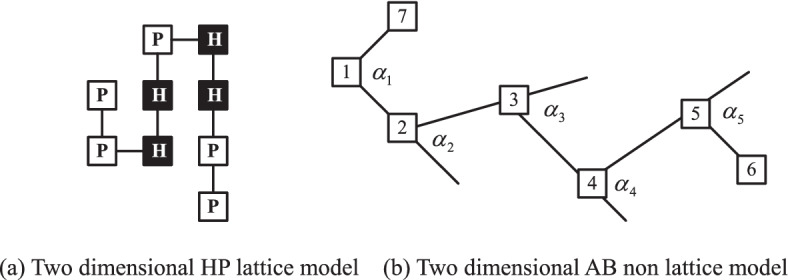
Two dimensional HP model AB model.

In the two-dimensional plane, the two-dimensional HP lattice model and the two-dimensional AB non lattice model are used to simulate the protein folding structure. The structure is as Fig. 3:

Figure 3a shows a two-dimensional HP lattice model. In the HP model, the amino acids are divided into hydrophobic amino acids and hydrophilic amino acids, which are represented by the English letters h and P respectively. The black square is hydrophobic amino acid h, and the white square is hydrophilic amino acid P. the distance between each interconnected amino acid is 1. And each lattice point must be placed on the integral point of the two-dimensional plane, that is, the angle between the interconnected amino acid residues cannot be changed. The black squares gather together, that is, the hydrophobic amino acids are concentrated in the hydrophilic amino acids, forming a hydrophobic core, which shows that the hydrophobic bond is used to maintain the stability of protein structure.

Figure 3b shows the two-dimensional AB non lattice model. Like HP lattice model, AB non lattice model also divides 20 kinds of amino acids into two categories, one is hydrophobic amino acids, which is represented by the letter A, the other is hydrophilic amino acids, which is represented by the letter B. However, compared with the two-dimensional HP lattice model, the angle between adjacent amino acids in AB non lattice model is variable, rather than fixed on the integer point of the two-dimensional plane. This kind of structure is more similar to the real spatial structure of protein, so it is more suitable to be used as the simulation structure of protein. In Fig. 3b, the white squares with numbers represent amino acids, and there will be an angle between the residues of two adjacent amino acids which can be represented by $\alpha_{1}$, $\alpha_{2}$, $\alpha_{3}$…

The energy function of two-dimensional AB non lattice model is expressed as follows:

1 $$E = \sum\limits_{i = 2}^{n - 1} {E_{1} (\alpha_{i} ) + } \sum\limits_{i = 1}^{n - 2} {\sum\limits_{j = i + 2}^{n} {E_{2} (r_{ij} ,\eta_{i} ,\eta_{j} )} }$$

here, $E$ represents the energy function; $n$ represents the number of amino acids; $E_{1}$ represents the potential energy function of the first part and expresses the energy contained in the folding of amino acid skeleton, which is related to each folding angle; $E_{2}$ represents the potential energy function of the second part and expresses the energy between non adjacent amino acid residues, which is related to the distance and polarity between residues.

By comparing the advantages and disadvantages of HP lattice model and ab non lattice model, AB non lattice model is used to express protein structure.

The stable structure of protein corresponds to the lowest energy value of protein potential energy function, which makes the prediction of protein stable structure into the optimization of energy function. In this field, tabu search and particle swarm optimization are common methods.

Because of its good memory function, tabu search algorithm has strong local search ability. By using tabu list and recording the searched local optimal solution, tabu search algorithm can avoid circuitous search and jump out of local optimal solution. At the same time, the tabu search algorithm can avoid missing good individuals by spurning some better solutions.

However, tabu search algorithm has a certain dependence on the initial solution. The better initial solution can make the tabu search algorithm find the better value, and the worse initial solution will reduce the convergence speed of tabu search algorithm.

Particle swarm optimization algorithm can get a better solution. Therefore, in this paper, the better solution obtained by particle swarm optimization algorithm is taken as the initial solution of tabu search algorithm, and then an optimization algorithm with both global search ability and local search ability is formed by using tabu strategy and contempt criterion of tabu search algorithm.

1. Generation of initial value

First, the particle swarm optimization algorithm is used to search globally. After converging to a certain extent, the better solution is used as the initial value of tabu search algorithm, so as to improve the search accuracy and efficiency of the algorithm.

The velocity and position of each particle are updated as shown in formula (2) and formula (3).

2 $$v_{id}^{k + 1} = \vartheta \times v_{id}^{k} + \lambda_{1} \times rand() \times (p_{id} - x_{id}^{k} ) + \lambda_{2} \times rand() \times (p_{gd} - x_{id}^{k} )$$

3 $$x_{id}^{k + 1} = x_{id}^{k} + v_{id}^{k + 1}$$

here, *v* is velocity; *x* is position; $\vartheta$ represents the inertia weight; $\lambda_{1}$ and $\lambda_{2}$ represents two learning factors; $rand()$ represents a random function; $p_{id}$ represents the historical optimal position of the ith particle; $p_{gd}$ represents the historical optimal position of the whole particle swarm.

(2) Neighborhood function

The neighborhood solution of tabu search algorithm is realized by single point mutation. In order to ensure the diversity of neighborhood solutions, this paper uses the silk line of disturbance variation to generate neighborhood solutions. Here, the neighborhood function is designed as follows:

4 $$x_{new}^{i} = x^{i} + f(q) \times \pi \times Q \times K$$

5 $$f(q) = \left\{ {\begin{array}{*{20}c} 1 & {q < 0.5} \\ { - 1} & {q \ge 0.5} \\ \end{array} } \right.$$

here $q$ and Q represents random number; $K$ represents the scale factor; $i$ denotes the generation times of neighborhood solutions. If the number of neighborhood solutions is $L$, then $i$ is the interval value of [0, L-1]. Here, set $K = 0.93$, $L = 40$.

(3) Candidate solution set

The candidate solution set is a subset of neighborhood solutions. In the algorithm, by calculating the fitness value of each of *L* neighborhood solutions, the first $L_{C}$ neighborhood solution with the lowest fitness value is selected as the candidate solution set. Here, set $L_{C} = 6$.

(4) Taboo list

Tabu list is used to store the recently searched solutions, and its length is $L_{T}$. After each iteration, the new tabu objects will enter the tabu list. At the same time, the term of office of each taboo object should be reduced by 1. Only when the term of office of the taboo object is 0 can the taboo be lifted, and the object who enters the taboo list first can be taboo out. The length of tabu list also has a certain influence on the search accuracy of the algorithm. If the length of tabu list is large, the search range of the algorithm is relatively wide, and a better solution can be found. But it also causes the search time of the algorithm to be longer and the convergence speed to be slower. If the length of the tabu list is too small, it can not play the role of the tabu list, and it is easy to make the algorithm into a circuitous search. Therefore, whether the length of tabu list is reasonable or not has a great impact on the results of the algorithm. Here, set $L_{T} = 8$.

(5) Taboo criterion

Let the fitness of the candidate solution $z(\alpha_{1} ,\alpha_{2} , \cdots ,\alpha_{n - 2} )$ be $E(z)$. There is a solution vector in tabu list $y(\alpha^{\prime}_{1} ,\alpha^{\prime}_{2} , \cdots ,\alpha^{\prime}_{n - 2} )$, and its fitness is $E(y)$. If the following conditions are met:

6 $$\left| {E(y) - E(z)} \right| \le E_{0}$$

7 $$\left\| {y - z} \right\| \le r_{0}$$

Then, the candidate solution z satisfies the tabu criterion, and the candidate solution is tabu.

Here, set $$E_{0} = 0.10$$, $$r_{0} = 0.005$$.

## Data Availability

The datasets used and analyzed during the current study are available from the corresponding author on reasonable request.

## Abbreviations

PSO: Particle swarm optimization
TS: Tabu search
NP: Nondeterministic polynomially
HP: Hydrophobic amino acid model
AB: Ashcroft-Bravais model
